# The DOT1L inhibitor Pinometostat decreases the host-response against infections: Considerations about its use in human therapy

**DOI:** 10.1038/s41598-019-53239-6

**Published:** 2019-11-14

**Authors:** Laura Marcos-Villar, Amelia Nieto

**Affiliations:** 1 Centro Nacional de Biotecnología (CNB-CSIC), Darwin 3, Cantoblanco 28049 Madrid, Spain; 2 CIBER de Enfermedades Respiratorias CIBERES, Madrid, Spain

**Keywords:** Cancer, Oncology, Cellular microbiology, RNA metabolism

## Abstract

Patients with acute myeloid leukemia frequently present translocations of *MLL* gene. Rearrangements of MLL protein (MLL-r) in complexes that contain the histone methyltransferase DOT1L are common, which elicit abnormal methylation of lysine 79 of histone H3 at *MLL* target genes. Phase 1 clinical studies with pinometostat (EPZ-5676), an inhibitor of DOT1L activity, demonstrated the therapeutic potential for targeting DOT1L in MLL-r leukemia patients. We previously reported that down-regulation of DOT1L increases influenza and vesicular stomatitis virus replication and decreases the antiviral response. Here we show that DOT1L inhibition also reduces Sendai virus-induced innate response and its overexpression decreases influenza virus multiplication, reinforcing the notion of DOT1L controlling viral replication. Accordingly, genes involved in the host innate response against pathogens (*RUBICON, TRIM25, BCL3*) are deregulated in human lung epithelial cells treated with pinometostat. Concomitantly, deregulation of some of these genes together with that of the *MicroRNA let-7B*, may account for the beneficial effects of pinometostat treatment in patients with MLL-r involving DOT1L. These results support a possible increased vulnerability to infection in MLL-r leukemia patients undergoing pinometostat treatment. Close follow up of infection should be considered in pinometostat therapy to reduce some severe side effects during the treatment.

## Introduction

Acute leukemia is the most frequent cancer and the leading cause of cancer mortality in the pediatric age. Two different types of leukemia have been recognized: acute lymphoblastic leukemia (ALL) and acute myeloid leukemia (AML), the former accounting for around 80% of all cases^1,2^. Chromosomal translocation is one of the main mechanisms responsible for tumor development and is very common in both types of leukemia^3,4^. One of the most common translocations involves the Mixed Lineage Leukemia (*MLL*) gene^3^; around 70% of infant leukemia and 10% of adult AML contain *MLL* rearrangements^5,6^.

Translocation of *MLL* gene generates new *MLL* fusion genes causing deregulation of genome expression and activation of oncogenes. More than 80 different partner genes have been described to fuse to *MLL* gene originating different protein complexes. Many of these complexes interact with factors involved in transcriptional regulation such as the histone methylase DOT1L that is frequently found in the complexes^6,7^. DOT1L protein is a methyltransferase that exclusively methylates lysine 79 of histone 3 (H3K79), adding mono- di- or trimethyl groups^8^. Some of the *MLL*-rearranged (MLL-r) leukemia genes are modulated through hypermethylation by DOT1L of a set of genes that elicit leukemogenesis. The pivotal role of DOT1L in MLL-r leukemia and its enzymatic specificity, have favored the search for chemical compounds directed against this molecule for use in leukemia treatment. Among DOT1L inhibitors, pinometostat has acquired a prominent role, since it constitutes a potent and specific inhibitor of DOT1L activity^9^. Treatment with pinometostat results in inhibition of H3K79 methylation and hence *MLL*-fusion genes expression^7,9,10^.

Influenza virus is an RNA virus whose transcription requires short-capped oligonucleotides as primers, which are scavenged from newly synthesized host RNA polymerase II transcripts,^11,12^. This transcription strategy requires, on-going cellular transcription, implies a functional association of virus transcription with host genome expression and thus depends on chromatin dynamic. Looking for epigenetic changes elicited by influenza virus infection, we observed specific changes in the methylation status of lysine 79 of histone 3 (H3K79) in infected human lung epithelial cells A549^13^. To characterize the possible contribution of H3K79 methylation to influenza virus control, treatment with pinometostat or/and use of specific lentiviral silencers for DOT1L expression were used. The data indicated that DOT1L down-regulates the antiviral signaling mediated by interferon and thus activates influenza virus replication through decrease of the antiviral response^13^. The effect of pinometostat controlling viral replication may be more general, since it also stimulates viral replication of vesicular stomatitis virus, a potent inducer of interferon^13^. From this study we concluded that methylation of H3K79 by DOT1L has an important role in the control of the interferon signaling and may modulate the infection of different pathogens.

## Results

### Effect of pinometostat in the control of interferon signaling

**Figure 1:**
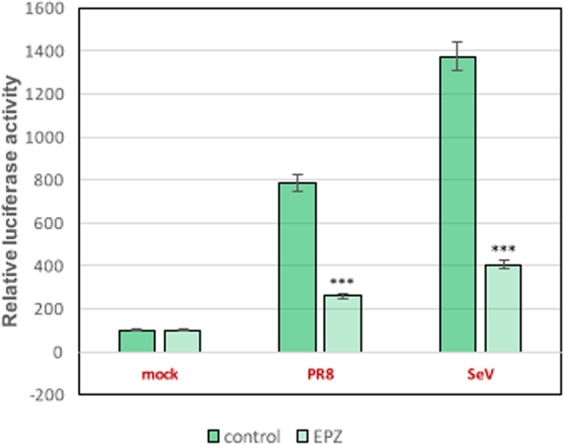
A549 cells untreated (Control) or treated with 1 μM pinometostat (EPZ) for 48 h, were transfected with plasmid pIF-lucter and infected with influenza virus or with Sendai virus at 1 pfu/cell. At 16 h post-infection, luciferase accumulation was monitored by luciferase Assay System (Promega). Three technical replicates of three independent experiments were analyzed. ns P > 0.05; *P < 0.05; **P < 0.01; ***P < 0.001.

Pinometostat treatment inhibits NF-κB nuclear translocation and reduces the expression of β-interferon and interferon stimulated genes such as Mx1 or ISG56 in influenza virus infected cells^13^. To further characterize pinometostat effects on IFN signaling, A549 cells untreated or treated with the DOT1L inhibitor for 48 h, were transfected with a plasmid expressing luciferase under the IFN-β promoter and infected at 1 plaque-forming units (pfu) per cell with influenza virus (PR8) or with Sendai virus (SeV), an efficient IFN-inducer virus^14^. At 16 h post-infection, luciferase accumulation was monitored as described in Materials and Methods (Fig. 1). Infection with either influenza or Sendai viruses stimulated luciferase accumulation in comparison with non-infected cells and pinometostat treatment caused a significant decrease of luciferase accumulation in the infected cells, reinforcing the role of DOT1L in controlling interferon signaling.

### DOT1L overexpression inhibits influenza virus replication

**Figure 2:**
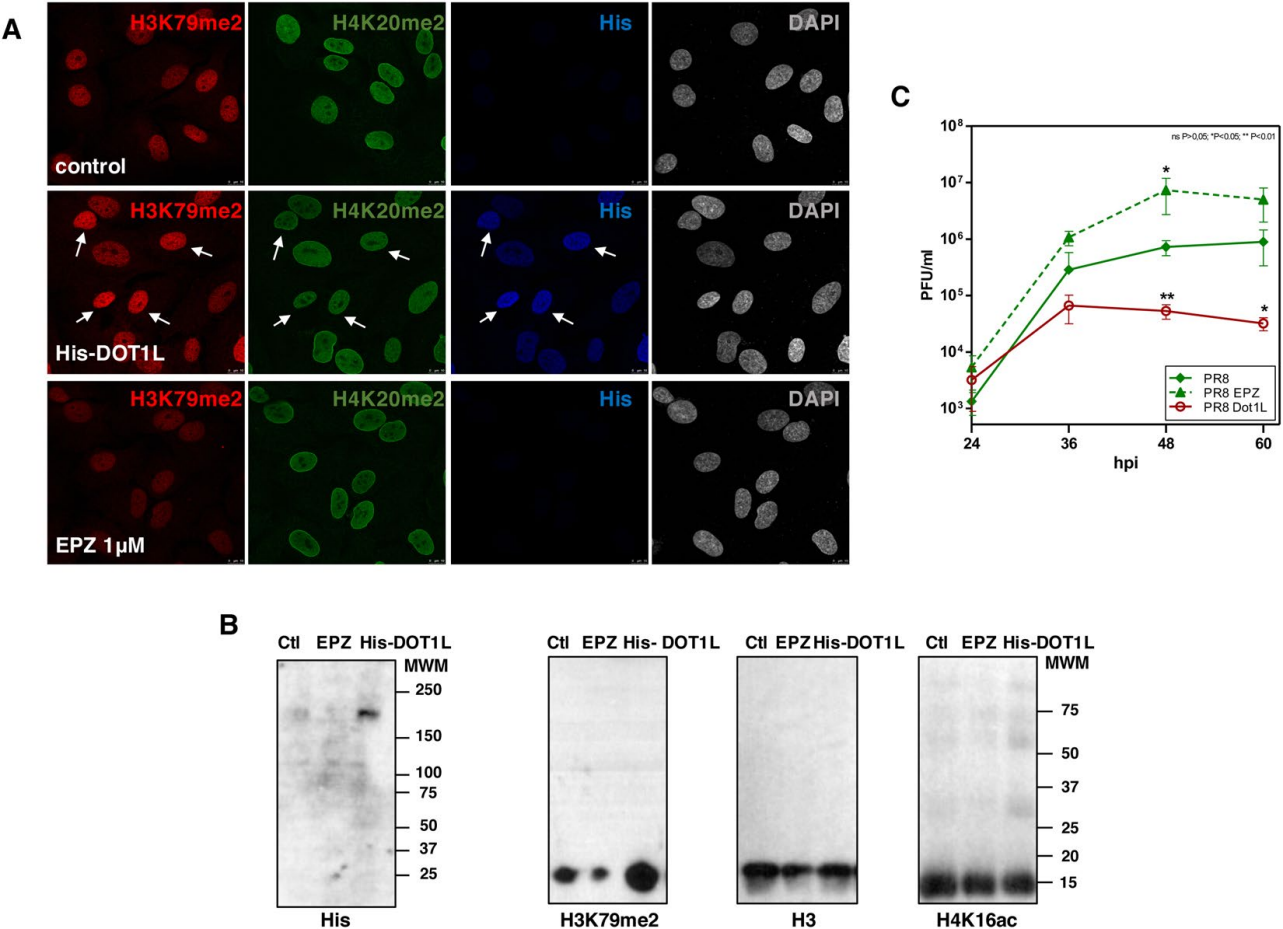
A549 untransfected cells (control), transfected with plasmid His-Dot1L 48 h (His-DOT1L) or treated with pinometostat 48 h (EPZ) were used (**A**) for immunofluorescence analysis against (His), H3K79me2 and H4K20me2. White arrows denote cells with high expression of His-DOT1L. (**B**) Western blots against His-DOT1L, H3K79me2, H3 and H4K16ac. (**C**) Untransfected cells (PR8), transfected with His-Dot1L 48 h (PR8 Dot1L) or treated with pinometostat 48 h (PR8 EPZ), were infected with 10^−3^ pfu/cell with PR8 influenza virus and infective particles analyzed by plaque assay. Three independent experiments were performed. ns P > 0.05; *P < 0.05; **P < 0.01; ***P < 0.001.

Down-regulation of DOT1L stimulates influenza and vesicular stomatitis virus replication in cultured cells^13^. The effect of DOT1L overexpression was now examined in A549 transfected cells with a plasmid that expresses human His-DOT1L protein. Pinometostat treated cells during 48 h (EPZ), untransfected cells (control or Ctl) or cells transfected with the plasmid that expresses His-DOT1L (His-DOT1L) during 48 h, were used to verify DOT1L overexpression and DOT1L enzymatic activity determined by the accumulation of H3K79me2, as analyzed by immunoflorescence (Fig. 2A) and Western blot (Fig. 2B). In addition, the accumulation levels of H4K20me2 were analyzed by immunofluorescence (Fig. 2A) and those of H3 and H4K16ac by Western blot as controls (Fig. 2B). Cells with up-regulated DOT1L expression were infected with influenza virus at 10^−3^ pfu/cell (PR8 Dot1L) and viral titters were analyzed by plaque assay at different times post-infection. In parallel, cells were treated with 1 μM pinometostat for 48 h and infected with PR8 under the same conditions (PR8 EPZ). An important and significant decrease in influenza virus replication was observed in A549 cells overexpressing DOT1L and a significant increase was apparent in pinometostat-treated cells (Fig. 2C).

### Effect of DOT1L downregulation in the expression of genes that control the host-response against infection in human lung epithelial cells

**Figure 3:**
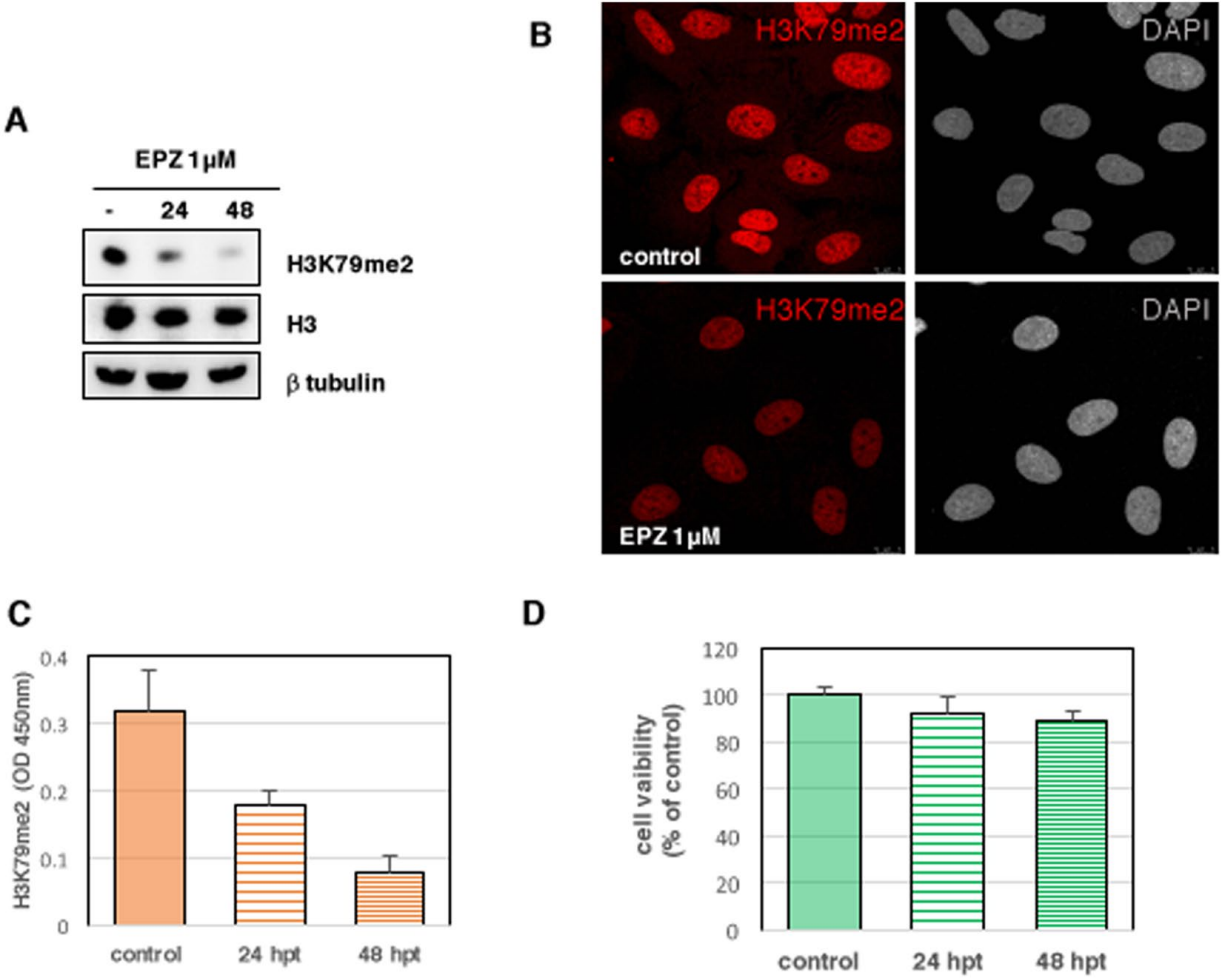
Total extracts of A549 cells untreated (control) or treated with pinometostat 1 μM (EPZ) during the indicated times were used for (**A**) Western blot against the specified proteins, (**B**) immunofluorescence analysis against H3K79me2 and (**C**) colorimetric detection of H3K79me2. (**D**) Viability of A549 cells treated with 1 μM DOT1L inhibitor was determined by MTT assay to measure cell metabolic activity.

Next we examined cellular targets methylated at H3K79 by performing RNA-seq analysis of A549 cells with active (control) or inactive DOT1L methylase (treated with 1 μM EPZ-5676, from Novagen). At various times post-treatment, the accumulation levels of methylated H3K79 were monitored by Western blot (Fig. 3A), immunofluorescence (Fig. 3B) and a quantitative colorimetric method based on anti-H3K79me2 antibody detection (Epigentek)^13^ (Fig. 3C). An important decrease on H3K79me2 levels was detected using all three different analyses, which indicate the efficiency of the pinometostat treatment. As previously showed^13^, treatment of A549 cells with 1μM pinometostat does not affect cell viability during 48 h, as analyzed by the MTT assay^15^ (Fig. 3D). RNAs isolated from duplicate cultures of untreated or treated cells during 48 h with pinometostat were used for high-throughput sequencing as described in Materials and Methods. Comparison of RNAs whose expression is modified during the treatment was performed using a minimum false-discovery rate (pvalue) of <0.001 as statistical significance and log2 fold change >2 as cut-off. Using these parameters, only a few RNAs changed in the presence of the inhibitor (Table 1), suggesting that H3K79 methylation occurs in a limited number of genes in these cells.

**Table 1:** List of proteins modulated by Pinometostat.

GENE NAME	Log2FC	DESCRIPTION
RUBCN	5.766742852	RUN and cysteine rich domain containing beclin 1 interacting protein
MIRLET7B	5.595201384	microRNA let-7b
PGBD2	4.909228246	piggyBac transposable element derived 2
TMEM181	4.758097693	transmembrane protein 181
SBNO1	4.547872227	strawberry notch homolog 1
DGCR8	4.43960124	DGCR8, microprocessor complex subunit
C15orf52	4.264753684	chromosome 15 open reading frame 52
REXO1	4.215797413	RNA exonuclease 1 homolog
TRIM25	3.50263504	tripartite motif containing 25
DMC1	3.188217042	DNA meiotic recombinase 1
TPR	−2.158545426	translocated promoter region, nuclear basket protein
BCL3	−2.963329088	B-cell CLL/lymphoma 3
NFATC2IP	−3.424029921	nuclear factor of activated T-cells 2 interacting protein
PRLH	−7.451413723	prolactin releasing hormone

Duplicate cultures of A549 cells were untreated or treated with pinometostat 1 μM, 48 h and total RNA was used for RNA-sequencing. The list of proteins encoded by the modulated genes using a qvalue <0.001 and log2 fold change >2 is shown.

In agreement with the role of H3K79 methylation in viral infection, alterations in *RUBCN, TRIM25* and *BCL3* gene expression in DOT1L down-regulated cells were found. In these conditions, expression of *RUBCN* and *TRIM25* was increased, while a reduction in the expression of *BCL3* was found.

- *RUBCN* gene encodes Rubicon, an important modulator of cellular autophagy; this is a fundamental pathway that eliminates intracellular pathogens through degradation^16^, activates intracellular signaling pathways and stimulates inflammatory mediators^17^. In addition, Rubicon works as negative regulator of type I interferon pathway through interacting with interferon regulatory factor 3 (IRF3)^18^. Down-regulation of Rubicon inhibits influenza and vesicular stomatitis virus replication due to promotion of type I IFN signaling and its overexpression causes the contrary effects^18^.
- Particular attention deserves changes in *TRIM25* expression. Thus, RIG-I is a sensor for viruses such as influenza A, Sendai virus, and flaviviruses^19–21^ and its activation is mediated by ubiquitination, which is partly mediated by TRIM25^22^. Accordingly, TRIM25 has been reported as a host restriction factor for HIV-1^23^ and influenza virus infection^22^.
- Bcl3 has been identified as an Ikkβ protein able to interacts with the p50 and p52 subunits of the NF-κB^24,25^. Bcl3 is a proto-oncogene that regulates cell proliferation and it is overexpressed in human T-cell leukemia virus infected cells eliciting overgrowth of the T infected cells^26^. It has been suggested that Bcl3 modulates NF-kB-dependent gene transcription^27,28^.

**Figure 4:**
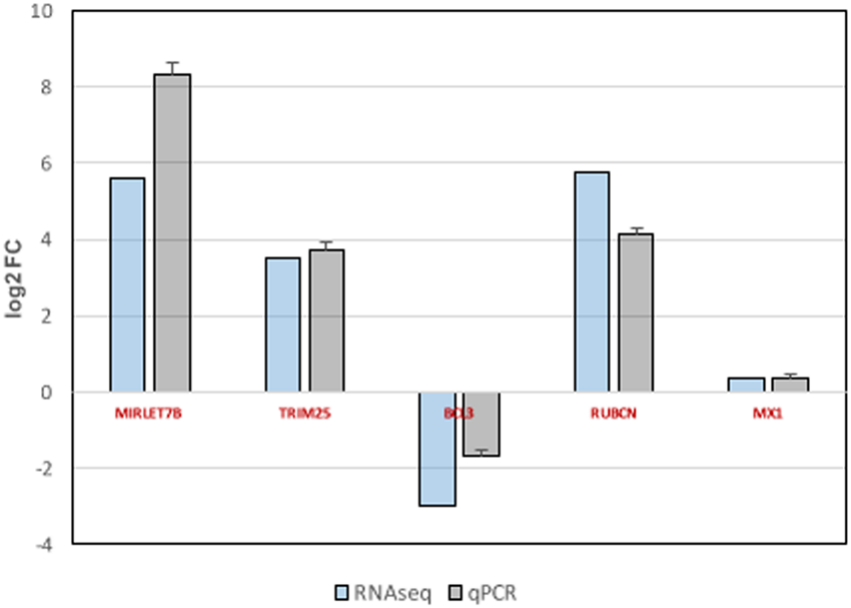
Comparison of RNA-seq and qRT-PCR data of A549 cells treated with pinometostat. For qRT-PCR analysis, A549 cells were plated alone or with pinometostat (1 μM,48 h). RNA was extracted and used for qPCR detection of MIRLET7B, TRIM25, BCL3, and RUBICON genes and MX1 as control. Comparison of the qRT-PCR and RNA-seq data is shown. The differences between untreated and treated cells were analyzed and are represented as log2 Fold Change. Three technical replicates of three independent experiments were performed.

To validate the RNA-seq results, the expression of these genes, as well as microRNA *let-7B* and an additional control gene not regulated by DOT1L (*Mx* gene) was evaluated by qRT-PCR analysis as described in Materials and Methods. Cultured A549 cells were left untreated or treated with 1 μM pinometostat for 48 h, total RNA was isolated and used for qRT-PCR studies. Comparison of the gene expression data obtained by the RNA-seq and qRT-PCR analysis is shown in Fig. 4, where similar values can be observed.

Together, our previous results^13^ and the RNA-seq experiments described here indicate that reduction of H3K79 methylation, alters the expression of several RNAs that encode proteins involved in the signaling pathways that control the host-response against pathogens.

## Discussion

Pinometostat was initially used in preclinical studies in animal models where it showed efficient eradication of leukemia clone from MLL-r leukemia^29^. Pinometostat also showed efficacy as antitumor drug in a xenograft model of MLL-r leukemia^10^ and has been the first member of histone methyltransferase inhibitors used in Phase 1 clinical trials in MLL-r patients, both adult and pediatric.

As shown in Table 1, pinometostat treatment alters the expression of some genes involved in leukemia control such as *MicroRNA let-7B* (let-7b) and *RUBICON* that are up-regulated, or *BCL3* that is down-regulated.

Expression of *let-7b* is remarkably decreased in ALL patients^30^, its potential role in diagnosis and its use as a therapeutic tool for infant ALL treatment have been suggested^31,32^. Down-regulation of DOT1L through pinometostat treatment notably increased *MicroRNA let-7b* expression in A549 cells (Table 1, Fig. 4). Thus pinometostat administration in patients could contribute to compensate the reduction of this micro RNA observed in ALL patients.

*RUBICON* expression is significantly decreased in AML cases compared with control group^33^. Accordingly, an involvement of autophagy pathway in the outcome of AML leukemia has been suggested. The increased expression of *RUBICON* in DOT1L down-regulated cells (Table 1, Fig. 4) could modulate autophagy, restricting the effect on proliferation of myeloid cells in pinometostat treated AML patients.

Treatment with pinometostat decreases *BCL3* gene expression (Table 1, Fig. 4). Bcl3 can exist as unphosphorylated form that functions as a conventional inhibitor of Ikkβ and promotes NF-κB activation^34^, and as an isoform phosphorylated by Akt, Erk2 or Ikk1/2 kinases^34^. Phosphorylation of Bcl3 promotes its nuclear translocation and its conversion into a transcriptional co-regulator of NF-κΒ, thus functioning as an oncoprotein^34^. Around 40% of AML patients have constitutively augmented activity of NF-κB and this activity seems to stimulate evasion of apoptosis and cell proliferation^35^. Also, AML CD34^+^ cells, express detectable activity of NF-κΒ^36^, reinforcing the notion that NF-κΒ signaling has a pivotal role in the development of AML leukemia. Inhibition of DOT1L induces an important decrease on *BCL3* expression, a gene with pivotal role controlling NF-κB activation.

Together, we show here that treatment of human epithelial cells with pinometostat modifies the expression of few cellular genes, being an important number of them involved in the modulation of leukemia altered pathways, such as the microRNA *let-7b*, *RUBICO*N, *or BCL3* genes.

The most common symptoms found in acute myeloid leukemia derive from alterations in blood cells levels, including decrease of neutrophils (neutropenia), which frequently causes fever as a symptom of subsequent infections. Different pathogens including gram negative and positive bacteria, viruses and fungi are responsible for the infection in patients with neutropenia (see for a review^37^) and the frequency and causative agent of the infections may depend on the treatment phase^38^.

Pinometostat has been used in Phase 1 clinical trials in MLL-r patients. In pediatric studies, treatment with pinometostat in 18 patients (from 3 months to 18 years of age) showed a transient reduction in peripheral or bone marrow blasts in 40% of the patients^39^. More than 20% of patients presented treatment-emergent adverse events (TEAEs) that included febrile neutropenia and respiratory failure among others^39^. In a recent survey of pinometostat treatment a total of 51 adult patients were engaged, 33 of which were AML patients with MLL-r and 4 ALL patients with MLL-r; the total number of patients with MLL-rearrangement represented 72.5% of the total cohort. Administration of pinometostat was safe, and its efficacy was modest, suggesting a possible therapeutic use of pinometostat when combined with other anti-leukemia agents^40^. The treatment-emergent adverse events of this study were febrile neutropenia (35%), cough (22%) and pneumonia (18%) and serious adverse effects were described in 35 patients (69%). The most common ones may be related with infection and were distributed as febrile neutropenia (25%), respiratory failure (12%) and pneumonia (10%)^40^.

NF-κB has a prominent role in the innate immune response against many different pathogens including bacteria, virus and fungi^41–44^. Activation of NF-κB is required to up-regulate apoptosis, Toll-like, RIG-I-like and NOD-like receptor signaling pathways^45^; which are common alterations shared in the host gene expression elicited by different relevant respiratory bacterial and viral pathogens^45^. Pinometostat treatment causes an important reduction on NF-κB nuclear translocation in cells activated with TNF-α or infected with influenza virus^13^. Hence, the serious adverse effects observed in MLL-r patients treated with pinometostat may be partly due to a decreased activation of NF-κB caused by the drug that would prevent the induction of innate host response and increase pathogen amplification.

### Future directions

Leukemia patients are particularly prone to pathogen infections. As shown above, pinometostat decreases signaling pathways that control the innate immune response against infections. Treatment with pinometostat is associated with a number of side effects that may derive from infections. Preventive administration of antibiotics or/and early survey of infections mediated by bacteria, virus or fungi should be considered concomitantly with pinometostat therapy to diminish severe side effects elicited by the treatment.

## Materials and Methods

### Western blotting

Western blotting was carried out as described previously^46^. The following antibodies were used: for H3K79me2 a rabbit polyclonal antibody (1:1000) (D15E8), for H3 a rabbit polyclonal antibody (1:1000) (D1H2), and for H4K16ac a rabbit polyclonal antibody (1:1000) (E2B8W) from Cell Signaling. For DOT1L detection, a rabbit polyclonal antibody anti-His (1:600) and for β-tubulin a mouse monoclonal antibody (1:1000), both from Sigma.

### Confocal immunofluorescence microscopy

A549 cells were used for immunofluorescence assays as described^13^ using a polyclonal antibody against H3K79me2 (1:250) from Cell Signaling, a polyclonal antibody against H4K20me2 (1:200) (NB100-66606) from Nobus and a monoclonal antibody anti-His (1:200) (631212) from Clontech for His-DOT1L detection with a Leica TCS SP5 laser scanning system.

### Transfection-infection experiments

A549 cells were left untransfected or transfected with a plasmid expressing His-DOT1L (Addgene) for 48 h. The cells were then infected with the A/Puerto Rico/8/34 (PR8) strain of influenza virus at 10^−3^ pfu/cell and virus multiplication was analyzed by plaque assay. Cells treated with 1 μM pinometostat (EPZ) for 48 h, and infected with PR8 at the conditions indicated above, were carried out in parallel. Three independent experiments were performed.

Human A549 cells were untreated (mock) or treated with 1 μM pinometostat (EPZ) for 48 h (EPZ), and then transfected with a luciferase reporter plasmid containing an IFN-β promoter (pIF-lucter), using VIROMER RED ONE. At 16 h post-transfection, cells were either mock infected or infected with PR8 or with Sendai virus (SeV) at 1 pfu/cell. At 16 h post-infection, luciferase accumulation was monitored using the luciferase Assay System (Promega). Three technical replicates of three independent experiments were analyzed.

In all transfection experiments, an amount of DMSO equivalent to the amount of this solvent in the pinometostat treated cells, was added to the untreated cells. A plasmid expressing renilla luciferase was added to the mixture in all transfection experiments to normalize transfection efficiency. Significance was determined by two way ANOVA analysis; (*P < 0.5; **P < 0.01; ***P < 0.001).

### RNA seq

Total RNAs were extracted using Trizol reagent (Invitrogen) according to the manufacturer’s instructions from control or pinometostat treated cells. Duplicate cultures of untreated or treated cells with 1 μM pinometostat during 48 h were used for high-throughput sequencing with TruSeq v3 chemistry and 50 bp single reads on an Illumina HiSeq. 2000. For RNA-seq analysis, sequenced reads were aligned to the Homo sapiens genome (version GRCh38.p8 from NCBI) using TopHat 2.1.1^47^ linked to Bowtie 2.2.8^48^ with default sensitive settings. From sequenced reads, transcripts were assembled using Cufflinks 2.2.1^49^ and differential expression analyses were performed with Cuffdiff 2.2.1^50^. Additional information for each gene was obtained from the NCBI database and included in the dataset. Genes with expression levels under a threshold in both control and treatment conditions were discarded. The median of the distribution of the non-zero values was taken as the threshold. The generated data were uploaded to the FIESTA viewer FIESTA@BioinfoGP for visualization and further filtering

### qRT-PCR analysis

For RNA extraction, cell pellets of untreated or EPZ-treated cells with 1 μM pinometostat during 48 h, were resuspended in 1 ml of TRIZOL reagent (Invitrogen) and the RNA was purified as recommended by the manufacturer. Reverse transcription was performed using the High-Capacity cDNA RT kit from Applied Biosystems. PCR was performed using SYBR green PCR master mix (Applied Biosystems) and specific primers.

## Data Availability

The datasets generated during the current study are deposited in Sequence Read Archive (SRA) of NCBI, with the following codes: BioProject; PRJNA562125, Study: SRP219262. https://dataview.ncbi.nlm.nih.gov/object/PRJNA562125?reviewer=bj3spmb63srg6bp6ab4j3ka78g.
